# IgM antiphospholipid antibodies are associated with a microvascular phenotype in antiphospholipid syndrome

**DOI:** 10.1186/s42358-026-00553-z

**Published:** 2026-06-11

**Authors:** Flavio Signorelli, Fernanda Oliveira de Andrade Lopes, Andreia Coimbra Sousa Gomes, Gustavo Guimarães Moreira Balbi, Irene Cecchi, Massimo Radin, Savino Sciascia, Danieli Castro Oliveira de Andrade

**Affiliations:** 1 Rheumatology Department, Hospital Universitário Pedro Ernesto, Universidade do Estado do Rio de Janeiro, Rio de Janeiro, Brazil; 2 Faculdade de Medicina, Hospital das Clínicas HCFMUSP, Universidade de São Paulo, Dr Arnaldo 455 Third Floor, Room 3192 - Cerqueira César, São Paulo, SP 05403-010 Brazil; 3 Rheumatology Service, Hospital Universitário da Universidade Federal de Juiz de Fora/EmpresaBrasileira de ServiçosHospitalares (HU-UFJF/Ebserh), Juiz de Fora, Minas Gerais Brazil; 4 Rheumatologic and Rare Diseases (ERK-Net, ERN-Reconnect and RITA-ERN Member) with Nephrology and Dialysis Unit and Center of Immuno-Rheumatology and Rare Diseases (CMID), Coordinating Center of the Interregional Network for Rare Diseases of Piedmont and Aosta Valley, San Giovanni Bosco Hub Hospital, Department of Clinical and Biological Sciences, University Center of Excellence on Nephrologic, University of Turin, Turin, Italy

**Keywords:** Antiphospholipid syndrome, Antiphospholipid antibodies, Lupus anticoagulant

## Abstract

**Background:**

The clinical significance of IgM antiphospholipid antibodies (aPL) in antiphospholipid syndrome (APS) remains uncertain, while lupus anticoagulant is a well-established marker for thrombotic risk.

**Methods:**

The objective of the study is to evaluate the impact of IgM aPL on the clinical phenotype of primary APS (PAPS). In this retrospective multicenter study, patients meeting updated Sapporo classification criteria were categorized into three aPL profiles: isolated lupus anticoagulant (isolated-LA), isolated IgM anticardiolipin and/or IgM anti-β2-glycoprotein I antibodies (isolated-IgM-aPL), and LA and IgM antibodies (LA + IgM-aPL). Clinical features were compared between isolated-LA vs. isolated-IgM-aPL and isolated-LA vs. LA + IgM-aPL groups.

**Results:**

Among 202 patients, 17 (8.4%) had isolated-IgM-aPL, 145 (71.7%) isolated-LA, and 40 (19.8%) LA + IgM-aPL. Compared to isolated-LA, the isolated-IgM-aPL group had lower female prevalence (41.2% vs. 65.5%; *p* = 0.049), fewer venous thromboses (41.2% vs. 66%; *p* = 0.049), but more obstetric morbidity (58.8% vs. 26.2%; *p* = 0.005). There was a higher proportion of patients with livedo racemosa (47.1% vs. 20.7%; *p* = 0.015) and with white matter lesions (WML) (29.4% vs. 9.7%; *p* = 0.020) in the isolated-IgM-aPL group. In the multivariate analysis, WML remained independently associated (OR 3.7; *p* = 0.020). In the second comparison (isolated-LA vs. LA + IgM-aPL), a higher prevalence of livedoid vasculopathy (15.0% vs. 4.8%; *p* = 0.026) and WML (22.5% vs. 9.7%; *p* = 0.037) were observed in the LA + IgM-aPL group. Nonetheless, no independent associations were seen in the multivariate analysis.

**Conclusion:**

IgM aPL may be associated with a distinct APS phenotype characterized by microvascular involvement, including livedo and WML. These findings support the need for further research into the clinical implications of IgM isotype positivity in APS.

## Introduction

Antiphospholipid syndrome (APS) is characterized by the presence of antiphospholipid antibodies (aPL) associated with arterial, venous, or microvascular thrombosis, pregnancy morbidity, or nonthrombotic manifestations [1]. APS can manifest as either a primary condition, occurring without an associated autoimmune disease, or as a secondary condition, which is linked to other autoimmune disorders, such as systemic lupus erythematosus (SLE) [2].

This wide range of clinical manifestations reflects the complexity of APS, which is further complicated by the variability in aPL profiles among affected individuals. The heterogeneous nature of these antibody profiles can influence the presentation and severity of APS, making diagnosis and management challenging [3, 4]. Among the aPL isotypes, lupus anticoagulant (LA) is the most well-established marker for thrombotic risk [5], whereas the clinical significance of IgM aPL remains a subject of ongoing debate [6]. The APS classification criteria, published in 2023, have introduced changes in how different aPL isotypes are weighted, reflecting the evolving understanding of their clinical relevance. Patients with isolated positivity to IgM isotypes do not fulfill the new criteria classification regardless of titers ≥ 40 units [1]. While IgG aPL are often emphasized due to its stronger association with thrombotic events, there is growing evidence suggesting that IgM aPL isotypes might also have important implications^3^. Recent studies have highlighted the distinct clinical subsets of thromboembolic events defined by IgM aPL, indicating that these antibodies might contribute significantly to APS [4]. Conversely, some researchers have proposed that IgM isotypes have weaker link to thrombosis and there is no added value for testing IgM-aPL in patients suspected of thrombotic APS [6]. This divergence in available evidence highlights a significant gap in the literature and underscores the need for further clarification and research.

Given the limited number of studies focusing specifically on the role of IgM aPL in APS, our study aims to analyze IgM aPL positive APS patients and compare them to LA positive APS patients to provide further insights on the impact of IgM aPL in the phenotypic expression of the disease. By that, we hope to contribute to the ongoing debate and enhance the understanding of APS subtypes and their management.

## Patients and data collection

We retrospectively included patients with primary APS who fulfilled the updated Sapporo classification criteria [7] from three referral centers up to November 2023. Patients were categorized into three distinct aPL profiles: isolated IgM antiphospholipid antibodies (isolated-IgM-aPL), isolated lupus anticoagulant (isolated-LA), and combined LA and IgM antibodies (LA + IgM-aPL).

Isolated-IgM-aPL was defined as the presence of IgM anticardiolipin (aCL) and/or IgM anti-β2 glycoprotein I (aβ2GPI) antibodies in the absence of all other aPLs, including IgG aCL, IgG aβ2GPI, and lupus anticoagulant. Patients were considered IgM-positive when aCL and/or aβ2GPI levels were ≥ 40 units, and high IgM titers were defined as ≥ 80 units [1]. Given that low to moderate levels of IgG aPL may be clinically relevant and influence patient classification, a cutoff of < 40 units was applied to define IgG negativity.

Similarly, isolated-LA was defined as the presence of lupus anticoagulant in the absence of any aCL or aβ2GPI antibodies. Other aPL profiles were excluded, as were patients with secondary APS. Lupus anticoagulant testing was performed according to ISTH guidelines [8].

After categorization, two main comparisons were performed regarding clinical data: (1) isolated-LA vs. isolated-IgM-aPL and (2) isolated-LA vs. LA + IgM-aPL. Patients were compared regarding demographic data (mean age, sex, race); comorbidities (hypertension, diabetes, obesity, dyslipidemia, stroke, myocardial infarction (MI); clinical manifestations: macrovascular events (venous or arterial), pregnancy morbidity, microthrombotic and non-thrombotic manifestations (livedo racemosa, livedoid vasculopathy, echocardiography-proven cardiac valve disease, nephropathy and thrombocytopenia) and white matter lesions (WML).

Recurrent arterial or venous thrombosis were defined as more than 1 event not occurring at the same time. Recurrent pregnancy morbidity was defined as: (1) at least 3 consecutive abortions or (2) more than one fetal loss or more than one premature birth before 34th week due to pre-eclampsia or placental insufficiency or (3) any combination of updated Sapporo obstetrics criteria. Comorbidities definitions are described elsewhere [9, 10, 11] and clinical manifestations and aPL positivity of APS patients were defined according to updated Sapporo classification criteria [7].

## Statistical analysis

Descriptive statistics were used to describe continuous variables (mean ± standard deviation - SD; median ± interquartile range - IQR) and categorical data were expressed as absolute number and percentage (%). The associations between clinical and laboratory variables in patients with isolated IgM isotype and those with other aPL antibodies were compared using the corrected χ² test and Fisher’s exact test for categorical data, and the Mann-Whitney U test for continuous variables. A significance level of 0.05 was applied. Odds-ratio (OR) were calculated with their 95% confidence interval (95% CI). A multivariate logistic regression analysis by Stepwise Backwards was performed to identify characteristics associated with IgM aPL profile; all variables with *p* < 0.1 in univariate analysis were included. All statistical analyses and Figures were conducted using SPSS software, version 26.0.

## Results

Two-hundred and two patients were assessed; of these, 17 (8.4%) had isolated-IgM-aPL, 145 (71.7%) had isolated-LA and 40 (19.8%) had LA + IgM-aPL. Female sex accounted for 121 (59.9%) of the patients, 141 (69.8%) were white and 182 (90.1%) had thrombosis. The median age of them was 47 (37–57) years-old. Main characteristics of the patients are shown in Table 1.

**Table 1:** Characteristics of 202 PAPS patients included in the study

Variables	*N*(%)
Age, years-old, (IQR)	47 (37–57)
Duration of disease, years-old (IQR)	15 (10–21)
Female Sex	121 (59.9%)
White race	141 (69.8%)
LA-isolated	145 (71.7%)
IgM-aPL-isolated	17 (8.4%)
LA + IgM-aPL	40 (19.8%)
Arterial hypertension	88 (43.6%)
Diabetes	21 (10.4%)
Obesity	64 (31.6%)
Dyslipidemia	87(43.1%)
Nephropathy	5 (2.5%)
Thrombocytopenia	16 (7.9%)
*Livedo racemosa*	49 (24.3%)
Valvopathy	19 (9.4%)
WML	28 (13.9%)
Livedoid vasculopathy	13 (6.4%)
Thrombosis	182 (90.1%)
Venous thrombosis	126 (62.4%)
Arterial thrombosis	87 (43.1%)
Stroke	51 (25.2%)
Obstetric APS	60 (29.7%)
Anticoagulant*	200 (99.0%)
Antiplatelet**	76 (37.6%)
Hydroxychloroquine	50 (24.8%)
Statins	75 (37.1%)

Legend: PAPS = primary antiphospholipid syndrome; LA= lupus anticoagulant; IgM = IgM anticardiolipin and/or IgM anti-β2-glycoprotein I antibodies; N= number; IQR= interquartile range; WML= white matter lesions. * Anticoagulant: warfarin (N-172) and low heparin molecular weight (*N* = 12); ** Antiplatelet: acetylsalicylic acid (*N* = 66) and clopidogrel (*N* = 10)

In comparison to isolated-LA, patients with isolated-IgM-aPL had lower prevalence of female sex (41.2% vs. 65.5%; *p* = 0.049), lower prevalence of venous thrombotic events (41% vs. 66%; *p* = 0.049) and higher prevalence of obstetric events (58.8% vs. 26.2%; *p* = 0.005). When we analyzed recurrent obstetric events, the IgM-aPL profile remained significant (*p* = 0.02). Patients from the IgM isolated-aPL group had a higher proportion of *livedo racemosa* (47.1% vs. 20.7%; *p* = 0.015) and WML (29.4% vs. 9.7%; *p* = 0.020), (Table 2). In the multivariate analysis, WML was significantly correlated with the isolated-IgM-aPL profile (B- Coefficient 1.32; Odds Ratio 3.7; 95% CI 1.15–12.2; *p* = 0.020).

**Table 2:** Comparison of primary PAPS patients with Isolated-LA vs. Isolated-IgM-aPL or LA + IgM-aPL

Variables	Isolated-LA (*N* = 145)^a^		Isolated-IgM-aPL (*N* = 17)^b^	LA + IgM-aPL (*N* = 40)^c^	*P* value^a, b^	*P* value^a, c^
Age, years-old (IQR)		46 (36.5–55.5)	45 (40–57)	50.5 (39.2–59.7)	0.928	0.109
Female Sex, n(%)		95 (65.5%)	7 (41.2%)	19 (47.5%)	**0.049**	**0.038**
White race, n(%)		100 (69.0%)	12 (70.6%)	29 (72.5%)	0.891	0.667
Arterial hypertension, n(%)		67 (46.2%)	4 (23.5%)	17 (42.5%)	0.075	0.677
Diabetes, n(%)		15 (10.3%)	0 (0.0%)	6 (15.0%)	0.371	0.411
Obesity, n(%)		49 (33.8%)	2 (11.8%)	13 (32.5%)	0.064	0.878
Dyslipidemia, n(%)		65 (44.8%)	8 (47.1%)	14 (35.0%)	0.861	0.266
Nephropathy, n(%)		5 (3.4%)	0 (0.0%)	0 (0.0%)	> 0.99	0.587
Thrombocytopenia, n(%)		12 (8.3%)	1 (5.9%)	3 (7.5%)	> 0.99	> 0.99
*Livedo racemosa*, n(%)		30 (20.7%)	8 (47.1%)	11 (27.5%)	**0.015**	0.359
Valvopathy, n(%)		13 (9.0%)	0 (0,0%)	6 (15.0%)	0.262	0.392
WML, n(%)		14 (9.7%)	5 (29.4%)	9 (22.5%)	**0.020**	**0.037**
Livedoid vasculopathy		7 (4.8%)	0 (0.0%)	6 (15.0%)	> 0.99	0.026
Venous thrombosis, n(%)		95 (65.5%)	7 (41.2%)	24 (60.0%)	**0.049**	0.519
Recurrent venous thrombosis, n(%)		40 (27.6%)	2 (11.8%)	7 (17.5%)	0.159	0.192
Arterial thrombosis, n(%)		66 (45.5%)	6 (35.3%)	15 (37.5%)	0.422	0.366
Recurrent arterial thrombosis, n(%)		28 (19.3%)	2 (12.5%)	5 (12.5%)	0.507	> 0.99
Stroke, n(%)		37 (25.5%)	5 (29.4%)	9 (22.5%)	0.729	0.696
Obstetric, n(%)*		38 (26.2%)	10 (58.8%)	12 (30.0%)	**0.005**	0.109
Recurrent obstetric, n(%)*		15 (16.5%)	7 (53.8%)	5 (20.8%)	**0.002**	**0.041**

Legend: PAPS = primary antiphospholipid syndrome; LA= lupus anticoagulant; IgM = IgM anticardiolipin and/or IgM anti-β2-glycoprotein I antibodies; n= number; IQR= interquartile range; WML= white matter lesions. *Exclusion of nulliparous and males: *N* = 91, *N* = 13 and *N* = 24 for LA-isolated, IgM-isolated and LA + IgM-aPL, respectively

Patients with combined positivity for LA + IgM-aPL were predominantly males (47.5% vs. 65.5%; *p* = 0.038), with higher prevalence of livedoid vasculopathy (15.0% vs. 4.8%; *p* = 0.026) and higher prevalence of WML (22.5% vs. 9.7%; *p* = 0.037) when compared to isolated-LA (Table 2). No independent associations were seen in the multivariate analysis.

**Fig. 1:**
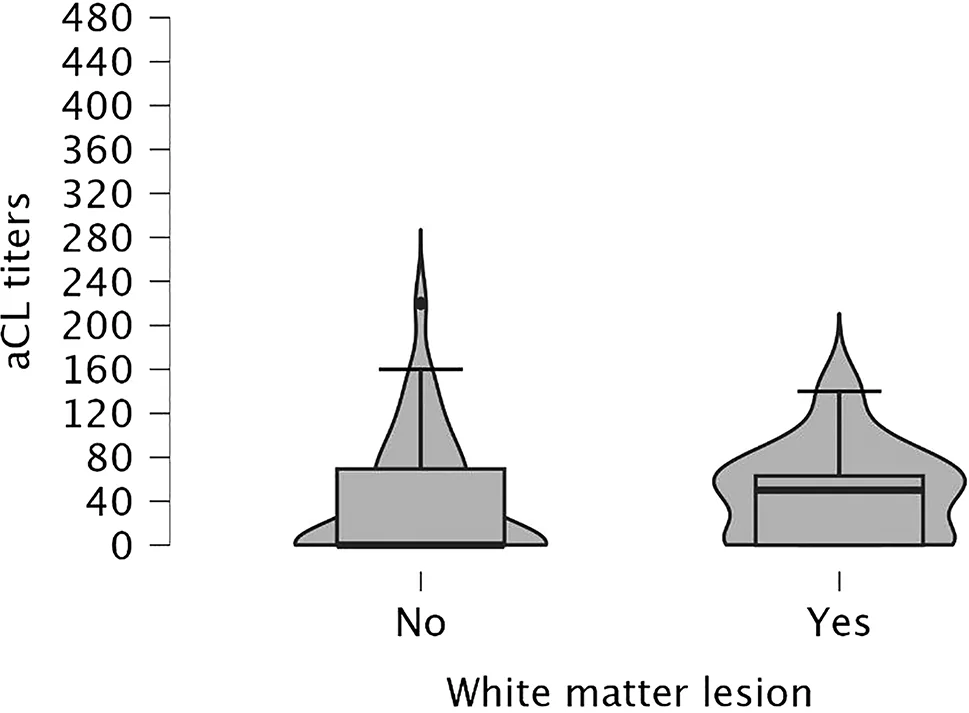
Correlation of white matter lesions (WML) and IgM anticardiolipin (aCL) titers

**Fig. 2:**
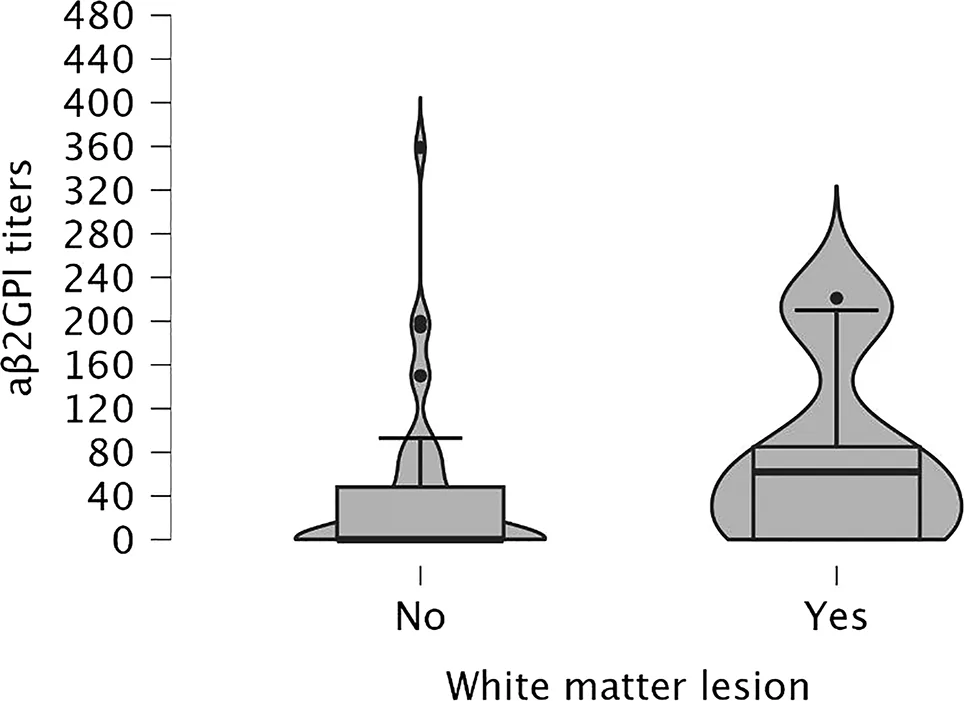
Correlation of white matter lesions (WML) and IgM anti-β2-glycoprotein I (aβ2GPI) titers

We also performed a subsequent analysis including all patients who tested positive for IgM, either isolated or associated with LA. When IgM isotype was analyzed as a continuous variable, increasing titers of both aCL IgM (*p* = 0.008) and anti-β2GPI IgM (*p* < 0.001) correlated with a higher prevalence of WML. The correlations between WML and IgM aCL and anti-β2GPI titers are shown in Figs. 1 and 2, respectively. These correlations were still observed after we categorized aCL IgM and anti-β2GPI IgM based on high titers (*p* = 0.02 and *p* < 0.001, respectively), ratifying the association between IgM and WML.

## Discussion

Isolated IgM antiphospholipid syndrome (APS) patients constituted 6.5% of thrombotic APS cases in our cohort, a prevalence consistent with previous reported ranges of 3.5% to 12.3%^6^. Despite this relative low percentage, the morbidity associated with thrombotic events underscores the importance of these findings. Even though thrombosis is more associated with the IgG than the IgM isotype, a minority of studies found significant associations with IgM but not with IgG antibodies [12].

Our findings suggest a lower prevalence of venous thrombosis in patients with isolated IgM-aPL compared to isolated LA, but not for arterial thrombosis. A retrospective study found a small but significant association between high-titer IgM aβ2GPI and recurrence arterial thrombosis, while venous recurrence was associated with aβ2GPI and aCL of the IgG isotypes [13].

We observed a notable higher prevalence of obstetric events in the isolated-IgM-aPL group compared to those with isolated-LA (58.8% vs. 26.2%; *p* = 0.005). This association aligns with previous studies that have established a relation between IgM aPL antibodies and adverse obstetric outcomes. For instance, Chayoua et al. performed a multicenter study involving 1,008 patients and found that isolated IgM was rare in thrombotic APS, but more frequent in obstetric APS, ranging from 3.5% to 5.4% and 5.7% to 12.3%, respectively. In the multivariate logistic regression analysis of aPL, IgM positivity was associated with pregnancy morbidity. Based on this data, the authors emphasize the potential benefit of screening for IgM aPL specifically in patients with obstetric complications [6]. This approach could improve early detection and management of APS in pregnant women, potentially reducing associated risks.

In our cohort we also observed an independent correlation between IgM aPL profile and WML. WML have recently been identified as more prevalent in APS patients with positive anti-neutrophil extracellular traps (NETs) IgG, adding another piece to the puzzle of APS pathogenesis [14]. The pathogenesis and etiology of WML is still unclear and one of the main contributors is known to be small vessel ischemic damage [15]. WML are considered to be a vascular contributor to various manifestations such as cognitive decline, dementia, depression, stroke and gait and balance problems. In a study performed by Urbanski et al., APS patients with isolated IgM anticardiolipin and/or aβ2GPI antibody had a higher rate of stroke, highlighting the macro thrombotic pathogenic role of this aPL. The positivity for isolated IgM anticardiolipin and/or aβ2GPI antibody remained an independent risk factor for stroke after adjustment for age, sex and cardiovascular risk factors [16]. Our study contributes to expanding the perception regarding not only macrovascular involvement, but also the potential relationship between IgM aPL and microthrombothic phenomena in the brain.

Moreover, a previous investigation reported a significant correlation between cognitive deficits and the presence of livedo racemosa and WML in APS patients [17]. Given that livedo racemosa and WML are both believed to be associated with microthrombotic events [1, 14], our findings showing a higher frequency of livedo racemosa (47.1% vs. 20.7%; *p* = 0.015) and with WML (29.4% vs. 9.7%; *p* = 0.020) in the isolated IgM-aPL group, support the hypothesis that IgM antibodies may be linked to a microthrombotic clinical phenotype. This aligns with the broader understanding that APS can manifest through various microvascular complications, including cognitive and neurological impairments.

When comparing patients with isolated-LA to those with LA + IgM-aPL, livedoid vasculopathy and white matter lesions (WML) appeared more frequent in the univariate analysis. However, these associations were not confirmed in the multivariate analysis. Notably, microvascular manifestations remained associated with the IgM aPL phenotype, independent of LA positivity.

Further research is needed to elucidate the mechanisms underlying these associations and to explore the potential benefits of early intervention strategies tailored to isolated IgM aPL patients. Understanding the full spectrum of clinical manifestations in this subgroup could lead to more effective management protocols and improved patient outcomes in APS.

Our research has limitations that must be considered when interpreting the results. First, this study has a retrospective design, which naturally restricts the ability to draw specific causal conclusions. Finally, since all patients derived from tertiary hospitals, the risk of selection bias and survivorship bias cannot be neglected. Conversely, this is the first study to suggest a correlation of microvascular manifestations linked to IgM-aPL. We also confirmed an important association with pregnancy morbidity.

## Conclusion

This study highlights a potential significance of antiphospholipid antibody isotype IgM in the phenotypic expression of PAPS. Despite the recommendations of predominantly focusing on IgG isotypes and LA due to their stronger association with thrombotic events, our findings suggest that IgM antibodies also play a significant role and should not be neglected in patients’ assessment. The impact of thrombotic APS, even in isolated IgM patients, highlights the necessity for ongoing research and clinical awareness for this subset of patients.

A particularly relevant discovery in this study was the association between IgM antibodies and WML, advocating a possible relation with microvascular phenomena.

Future investigations should explore in more detail the mechanisms by which IgM antibodies contribute to APS pathogenesis and how they can be better integrated into clinical practice to optimize patient management. 

## Data Availability

The data underlying this article cannot be shared publicly for the privacy of individuals that participated in the study]. The data will be shared on reasonable request to the corresponding author.

## Abbreviations

aβ2GPI: Anti-β2-glycoprotein I antibodies
aCL: Anticardiolipin antibodies
aPL: Antiphospholipid antibodies
APS: Antiphospholipid syndrome
CI: Confidence interval
IQR: Interquartile range
Isolated-IgM-aPL: Isolated IgM anticardiolipin and/or IgM anti-β2-glycoprotein I antibodies ≥40 units
Isolated-LA: Isolated lupus anticoagulant
ISTH: International Society on Thrombosis and Haemostasis
LA: Lupus anticoagulant
LA+IgM-aPL: LA and IgM antibodies ≥40 units
MI: Myocardial infarction
NETs: Neutrophil extracellular traps
OR: Odds ratio
SD: Standard deviation
SLE: Systemic lupus erythematosus
SPSS: Statistical Package for the Social Sciences
WML: White matter lesions

## References

[1] Barbhaiya M, Zuily S, Naden R, Hendry A, Manneville F, Amico MC, et al. 2023 ACR/EULAR antiphospholipid syndrome classification criteria. Ann Rheum Dis. 2023;82:1258–70. 10.1136/annrheumdis-2023-224123. 37640450 10.1136/ard-2023-224609

[2] Dabit JY, Valenzuela-Almada MO, Vallejo-Ramos S, Duarte-García A. Epidemiology of Antiphospholipid Syndrome in the General Population. Curr Rheumatol Rep. 2022;23:85. 10.1007/s11926-022-01099-2. 34985614 10.1007/s11926-021-01038-2 PMC8727975

[3] Del Ross T, Ruffatti A, Cuffaro S, Tonello M, Calligaro A, Favaro M, et al. The clinical relevance of the IgM isotype of antiphospholipid antibodies in the vascular antiphospholipid syndrome. Thromb Res. 2015;136:883–6. 10.1016/j.thromres.2015.08.019. 26410418 10.1016/j.thromres.2015.08.019

[4] Locht H, Wiik A. IgG and IgM isotypes of anti-cardiolipin and anti-beta2-glycoprotein I antibodies reflect different forms of recent thrombo-embolic events. Clin Rheumatol. 2006;25:246–50. 10.1007/s10067-005-0044-6. 16177835 10.1007/s10067-005-1166-x

[5] Gebhart J, Posch F, Koder S, Perkmann T, Quehenberger P, Zoghlami C, et al. Increased mortality in patients with the lupus anticoagulant: the Vienna Lupus Anticoagulant and Thrombosis Study (LATS). Blood. 2015;125:3477–83. 10.1182/blood-2014-11-612746. 25810488 10.1182/blood-2014-11-611129

[6] Chayoua W, Kelchtermans H, Gris JC, Moore GW, Musiał J, Wahl D, et al. The (non-)sense of detecting anti-cardiolipin and anti-β2glycoprotein I IgM antibodies in the antiphospholipid syndrome. J Thromb Haemost. 2020;18:169–79. 10.1111/jth.14635. 31519058 10.1111/jth.14633

[7] Miyakis S, Lockshin MD, Atsumi T, Branch DW, Brey RL, Cervera R, et al. International consensus statement on an update of the classification criteria for definite antiphospholipid syndrome (APS). J Thromb Haemost. 2006;4:295–306. 10.1111/j.1538-7836.2006.01753.x. 16420554 10.1111/j.1538-7836.2006.01753.x

[8] Devreese KMJ, Bertolaccini ML, Branch DW, de Laat B, Erkan D, Favaloro EJ, et al. An update on laboratory detection and interpretation of antiphospholipid antibodies for diagnosis of antiphospholipid syndrome: guidance from the ISTH-SSC Subcommittee on Lupus Anticoagulant/Antiphospholipid Antibodies. J Thromb Haemost. 2025;23:731–44. 10.1111/jth.14918. 39510414 10.1016/j.jtha.2024.10.022

[9] Grundy SM, Stone NJ, Bailey AL, Beam C, Birtcher KK, Blumenthal RS, et al. 2018 AHA/ACC/AACVPR/AAPA/ABC/ACPM/ADA/AGS/APhA/ASPC/NLA/PCNA Guideline on the Management of Blood Cholesterol: A Report of the American College of Cardiology/American Heart Association Task Force on Clinical Practice Guidelines. J Am Coll Cardiol. 2019;139:e1082–e1143. 10.1016/j.jacc.2018.11.003. 10.1161/CIR.0000000000000625 PMC7403606 30586774

[10] American Diabetes Association Professional Practice Committee; 2. Classification and diagnosis of diabetes: standards of medical care in diabetes—2022. Diabetes Care. 2022;45:S17–S38. 10.2337/dc22-S002. 10.2337/dc22-S002 34964875

[11] Whelton PK, Carey RM, Aranow WS, Casey DE Jr, Collins KJ, Dennison Himmelfarb C, et al. 2017 ACC/AHA/AAPA/ABC/ACPM/AGS/APhA/ASH/ASPC/NMA/PCNA Guideline for the prevention, detection, evaluation, and management of high blood pressure in adults: a report of the American College of Cardiology/American Heart Association Task Force on Clinical Practice Guidelines. Hypertension. 2018;71:e13–e115. 10.1161/HYP.0000000000000065. 10.1161/HYP.0000000000000065 29133356

[12] Kelchtermans H, Pelkmans L, de Laat B, Devreese KM. IgG/IgM antiphospholipid antibodies present in the classification criteria for the antiphospholipid syndrome: a critical review of their association with thrombosis. J Thromb Haemost. 2016;14:1530–48. 10.1111/jth.13379. 27279342 10.1111/jth.13379

[13] Niznik S, Rapoport MJ, Avnery O, Lubetsky A, Haj Yahia S, Ellis MH, Agmon-Levin N. Patterns of Recurrent Thrombosis in Primary Antiphospholipid Syndrome—Multicenter, Real-Life Long-Term Follow-Up. Front Immunol. 2022;13:843718. 10.3389/fimmu.2022.843718. 35514968 10.3389/fimmu.2022.843718 PMC9063725

[14] Zuo Y, Navaz S, Tsodikov A, Kmetova K, Kluge L, Ambati A, et al. Anti–Neutrophil Extracellular Trap Antibodies in Antiphospholipid Antibody–Positive Patients: Results From the Antiphospholipid Syndrome Alliance for Clinical Trials and InternatiOnal Networking Clinical Database and Repository. Arthritis Rheumatol. 2023;75:1407–14. 10.1002/art.42489. 36862141 10.1002/art.42489 PMC10758259

[15] Frey BM, Petersen M, Mayer C, Schulz M, Cheng B, Thomalla G. Characterization of White Matter Hyperintensities in Large-Scale MRI-Studies. Front Neurol. 2019;10:238. 10.3389/fneur.2019.00238. 30972001 10.3389/fneur.2019.00238 PMC6443932

[16] Urbanski G, Yelnik CM, Maillard H, Launay D, Dubucquoi S, Hachulla E, et al. Antiphospholipid Syndrome With Isolated Isotype M Anticardiolipin and/or Anti-B2GPI Antibody Is Associated With Stroke. Stroke. 2018;49:2770–2. 10.1161/STROKEAHA.118.023021. 30355196 10.1161/STROKEAHA.118.023021

[17] Tektonidou MG, Varsou N, Kotoulas G, Antoniou A, Moutsopoulos HM. Cognitive Deficits in Patients With Antiphospholipid Syndrome: Association With Clinical, Laboratory, and Brain Magnetic Resonance Imaging Findings. Arch Intern Med. 2006;166:2278–84. 10.1001/archinte.166.20.2278. 17101948 10.1001/archinte.166.20.2278

